# Low back pain, occupational stress, and physical fitness in intensive care nursing professionals

**DOI:** 10.1590/0034-7167-2024-0583

**Published:** 2026-08-17

**Authors:** Ulisses Masseli Dias, Silvio Assis de Oliveira-Junior, Jeeser Alves de Almeida, Paula Felippe Martinez

**Affiliations:** I Universidade Federal de Mato Grosso do Sul. Campo Grande, Mato Grosso do Sul, Brazil; II Empresa Brazileira de Serviços Hospitalares. Campo Grande, Mato Grosso do Sul, Brazil

**Keywords:** Low Back Pain, Physical Fitness, Occupational Stress, Nurses, Intensive Care Units., Dolor de la Región Lumbar, Aptitud Física, Estrés Laboral, Enfermeras y Enfermeros, Unidades de Cuidados Intensivos.

## Abstract

**Objectives:**

to assess the association between low back pain, occupational stress, and physical fitness in intensive care nursing professionals.

**Methods:**

quantitative cross-sectional study with convenience sampling, including sociodemographic data, occupational stress, and physical fitness (cardiorespiratory capacity, body composition, flexibility, strength, and muscle endurance). Low back pain was assessed using disability, duration, and frequency scales.

**Results:**

among 83 intensive care nursing professionals, 72.3% reported low back pain in the past year, and 50.6% presented with moderate to high occupational stress. Low back pain was associated with workload and trunk flexor strength. The other physical fitness variables did not vary among groups. Functional disability was similar among individuals with and without low back pain.

**Conclusions:**

trunk flexor strength was associated with a lower low back pain incidence in intensive care nursing professionals. Vigorous physical activity showed a tendency towards association, while occupational stress did not show a relationship with low back pain.

## RESUMEN

**Objetivos:**

evaluar la asociación entre el dolor lumbar, el estrés laboral y la aptitud física en profesionales de enfermería de cuidados intensivos.

**Métodos:**

estudio transversal cuantitativo con muestreo por conveniencia, que incluyó datos sociodemográficos, estrés laboral y aptitud física (capacidad cardiorrespiratoria, composición corporal, flexibilidad, fuerza y resistencia muscular). El dolor lumbar se evaluó mediante escalas de discapacidad, duración y frecuencia.

**Resultados:**

entre 83 profesionales de enfermería de cuidados intensivos, el 72,3 % reportó dolor lumbar en el último año y el 50,6 % presentó estrés laboral de moderado a alto. El dolor lumbar se asoció con la carga de trabajo y la fuerza de los flexores del tronco. Las demás variables de aptitud física no variaron entre los grupos. La discapacidad funcional fue similar entre individuos con y sin dolor lumbar.

**Conclusiones:**

la fuerza de los flexores del tronco se asoció con una menor incidencia de dolor lumbar en profesionales de enfermería de cuidados intensivos. La actividad física vigorosa mostró una tendencia a la asociación, mientras que el estrés laboral no mostró relación con el dolor lumbar.

## INTRODUCTION

Lower back pain is a prevalent condition among healthcare professionals, especially among nurses in Intensive Care Units (ICUs), with studies revealing an occurrence between 76% and 80.5%, frequently associated with high workloads and physically strenuous activities^(1,2)^. Concomitantly, occupational stress is frequent among ICU nurses, with moderate levels predominating (87%), and low (11.7%) or high (1.3%) levels being less common^(3)^.

Conversely, health-related physical fitness, defined as the ability to perform daily physical activities with vigor and without excessive fatigue, including components such as muscle strength, cardiorespiratory capacity, flexibility, and body composition^(4)^, emerges as a potentially protective factor, positively influencing healthcare professionals’ quality of life and well-being. Regular physical activity has been associated with reduced perceived stress and improved mental well-being in healthcare professionals, although more studies are needed to elucidate the extent of these benefits^(5)^.

Specific components of physical fitness, such as strength, endurance, and flexibility, can contribute to reducing lower back pain and occupational stress among nurses. Muscle strengthening and flexibility help prevent musculoskeletal pain, while endurance promotes adaptation to physical and psychological stress, fostering greater resilience in hospital settings^(6)^. Furthermore, exercise programs that focus on muscle strengthening and physical capacity can reduce the intensity of lower back pain in nursing professionals, contributing to occupational disability prevention^(7)^.

Despite evidence of the benefits of physical fitness in mitigating low back pain and occupational stress, the specific relationships among these factors in ICU nurses are still poorly explored, requiring more detailed studies.

Understanding these interactions is essential for developing strategies aimed at promoting intensive care nursing professionals’ (ICNPs) health and well-being, strengthening both workplace safety and patient care quality. Identifying risk and protective factors allows for more precise interventions, contributing to musculoskeletal disorder prevention and improving these professionals’ quality of life.

## OBJECTIVES

To assess the association between low back pain, occupational stress, and health-related physical fitness in active and inactive ICNPs at a university hospital.

## METHODS

### Ethical aspects

The study was conducted in accordance with the ethical principles of Resolution 466/2012 of the Brazilian National Health Council and Resolution CNS 580/2018, which regulate institutional approval and non-interference in the routine of services. Institutional authorization was obtained through an administrative resolution, which mandates the citation of the *Hospital Universitário Maria Aparecida Pedrossian* of the *Universidade Federal de Mato Grosso do Sul* (HUMAP-UFMS) in all scientific publications of the project. The project was approved by the UFMS Research Ethics Committee, and all participants signed the Informed Consent Form.

### Study design, period and location

This observational, analytical, cross-sectional, and quantitative study used non-probabilistic convenience sampling, and was reported in accordance with STrengthening the Reporting of OBservational studies in Epidemiology guidelines^(8)^. Data collection was carried out between June and July 2024 at HUMAP, located in the Central-West region of Brazil, linked to UFMS and managed by the *Empresa Brasileira de Serviços Hospitalares* (Ebserh).

### Population and sample; inclusion and exclusion criteria

**Figure 1:**
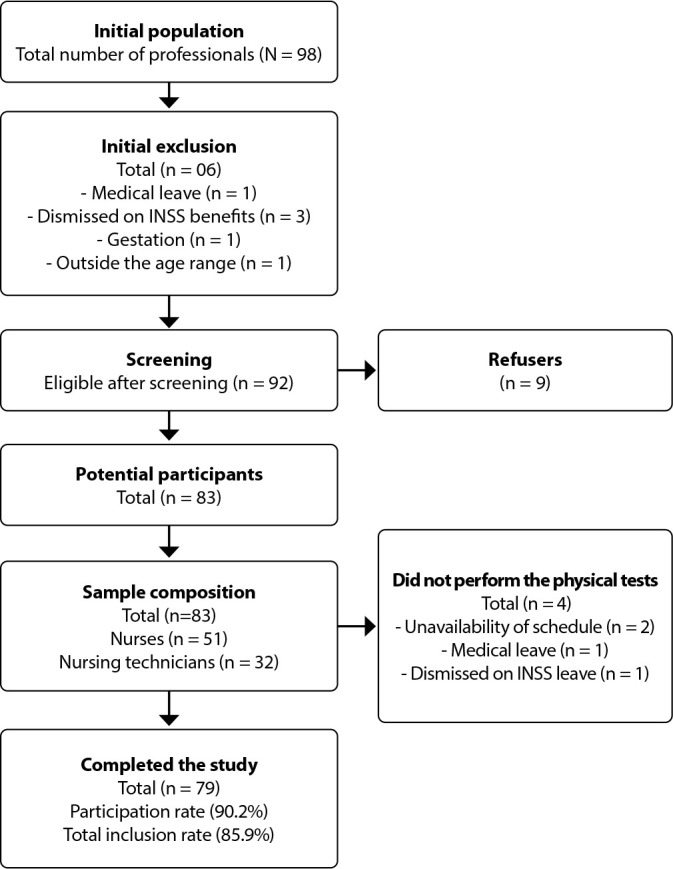
Flowchart for participant selection for a study with intensive care nursing professionals, Campo Grande, Mato Grosso do Sul, Brazil, 2024

The study population consisted of 98 ICNPs, comprised of nurses and nursing technicians working in the Adult ICU, Pediatric ICU, and Neonatal ICU of a university hospital. Since the entire target population was analyzed, sample size calculation was not necessary. Inclusion criteria included ICNPs in good health, physically capable of participating in the study activities, aged between 18 and 55 years, and without restrictions regarding sex, color, or race/ethnicity. Initial screening was conducted using a clinical-occupational questionnaire designed to assess health status and physical condition, with items on illnesses, surgeries, and eligibility, and supplemented by validated instruments: a physical activity questionnaire; a disability questionnaire due to low back pain; and an occupational stress scale. Good health and physical capacity were confirmed by self-report in the initial questionnaire, which verified the absence of systemic diseases, spinal surgeries, disabilities, or medical leave, as well as the ability to perform physical tests without medical contraindications, with no requirement for normality indices. ICNPs with low back pain associated with systemic diseases, pregnant women or women up to six months postpartum, a history of lumbar spine surgery, degenerative joint diseases, disabling disabilities, and those on medical leave or absence were excluded. Six participants were excluded based on the defined exclusion criteria, and nine did not agree to participate. The sample consisted of 51 nurses and 32 nursing technicians, distributed among the Adult ICU, Pediatric ICU, and Neonatal ICU, with ages ranging from 27 to 55 years. In total, 79 ICNPs completed the study, while four participants did not complete the second stage, resulting in a participation rate of 90.2%, as shown in Figure 1.

*INSS - Brazilian National Institute of Social Security.*

*Note: all stages of the study were completed by 79 participants. Nine opted not to participate; six were excluded due to ineligibility; and four did not complete the physical tests. Metrics: participation rate (90.2%) = [(83 / 92) × 100 ≈ 90.2%], based on potential participants (n = 83) and eligible participants after screening (n = 92); total inclusion rate (85.9%) = [(79 / 92) × 100 ≈ 85.9%] based on those who completed the study (n = 79) and eligible participants after screening (n = 92).*

### Study protocol and variables

Data collection was carried out in two stages: (1) Sociodemographic, Occupational and Clinical Questionnaire (SOQC), applied online (Google Forms^®^) covering sociodemographic, occupational and clinical data, including the International Physical Activity Questionnaire (IPAQ)^(9)^, the Roland-Morris Disability Questionnaire (RMDQ)^(10)^ and the Bianchi Stress Scale (BSS)^(11)^; and (2) conducting physical fitness assessments, in scheduled sessions, aimed at collecting health and physical fitness data, including Body Mass Index (BMI)^(12)^, body fat (BF) percentage using the Jackson and Pollock seven-fold method^(13)^, handgrip strength (HGS)^(12)^, flexibility using the sit-and-reach test (SRT)^(14)^, trunk muscle endurance strength using the Ito test^(15)^ and cardiorespiratory assessment using the Ruffier-Dickson test (RDT)^(16)^.

The QSOC collected information on sex, age, race/ethnicity, religious beliefs, educational level, family income, job title, work shift and weekly hours, length of experience in general nursing and intensive care nursing, existence of other employment, and associated activities. Lower back pain duration and frequency in the last 12 months were also assessed, with its presence classified into five groups: absence of pain, recurrent episodes, acute pain (≤ 6 weeks), subacute pain (6-12 weeks), and chronic pain (> 12 weeks), based on duration and frequency^(17)^. For statistical analysis, low back pain was dichotomized into presence (any report in the last 12 months) and absence (no pain).

Disability related to low back pain was measured using the RMDQ^(10,18)^, which scores from 0 (no disability) to 24 (severe disability). For severity stratification, the following categorization was adopted: mild (0-8), moderate (9-16), and high (17-24)^(19)^. For analytical purposes, functional disability was dichotomized into mild (≤ 8 points) and moderate/high (> 8 points), seeking greater interpretative clarity and statistical consistency.

Occupational stress was analyzed by the BSS^(11)^, with 51 items, organized into six domains, using the total score, dichotomized cut-off point ≤ 3, for “low”, and > 3, for “medium/high”.

Additionally, the level of physical activity was assessed using the IPAQ short version, validated in Brazil^(9)^. The IPAQ analyzes the time spent on walking, moderate and vigorous activities, and sedentary behavior. Based on results, individuals were categorized into two groups: active, which includes the “very active” and “active” categories, with adequate levels of physical activity; and somewhat active, which encompasses the “insufficiently active” and “sedentary” categories, representing insufficient levels of physical activity or a lack of regular involvement in exercise. BMI was calculated by the ratio between body mass (kg) and height squared (m^2^)^(12)^. In the study, body mass was measured using a G-Tech electronic scale (Glass 3FWB, 100 g precision), and height was measured using a BIC brand portable digital stadiometer (0.1 cm precision)^(20)^. The BMI classification was dichotomized into BMI ≥ 25 kg/m^2^ (high risk) and BMI < 25 kg/m^2^ (low risk).

Regarding the components of physical fitness related to health, body composition, estimated by BF percentage, which constitutes a risk indicator for metabolic, cardiovascular and psychosocial diseases^(21)^, was assessed by skinfold thickness at seven anatomical points with the Opus Max scientific caliper, and calculated using the Jackson and Pollock formulas (1978)^(13)^. HGS, an indicator of overall muscle strength^(22)^ and a variable associated with the presence of low back pain^(23)^, was assessed with the participant seated, considering the grip strength of the dominant hand, measured with a Jamar hydraulic dynamometer, in three trials, with a one-minute interval between them^(12)^, and the results were compared with classifications by sex and age group^(24)^. RST, which assesses the flexibility of the posterior chain^(13)^, was performed with the participant seated on a stable surface covered with a thin mat (1.5 cm thick), with knees extended and feet fixed without shoes, flexing the trunk forward until reaching the greatest possible distance with the fingers. Using a portable Wells bench (Prime Med), the test was performed in three attempts, considering the greatest distance reached from the middle finger, and the results were compared with the Pollock ML and Wilmore JH classification (1993)^(25)^. The strength and endurance of the trunk flexor and extensor muscles was assessed using the Ito test^(15)^, which uses isometric flexion and extension postures performed in prone and supine positions until participants’ resistance limit (maximum of five minutes). Time was recorded with a digital stopwatch.

Finally, cardiorespiratory fitness was assessed using the RDT, which consists of performing 30 squats in 45 seconds^(16,20,26,27)^, a clinically validated test associated with reduced cardiovascular risk and increased VO_2_ max^(26,27)^. Posture was adjusted to ensure 90° knee flexion, and heart rate (P1, P2, and P3) was measured using the Samsung Gear S3 Frontier smartwatch. The rhythm of the 30 squats was controlled at 40 movements per minute (bpm) using the metronome from the Soundcorset Tuner & Metronome^®^ app, and the results were interpreted using the Ruffier Index^(20,27)^.

### Statistical analysis

Data collected via Google Forms^®^ were analyzed using Jamovi version 2.6 (Sydney, Australia). They were presented as descriptive measures, including mean and standard deviation, median and interquartile range, as well as absolute and relative frequencies. Categorical variables were assessed using Fisher’s exact test or the chi-square test (χ^2^), while the normality of continuous variables was analyzed using the Shapiro-Wilk test. The association between physical fitness and physical activity level was tested by comparing active and less active groups, using Student’s t-test for normally distributed variables and the Mann-Whitney test for non-parametric distributed variables. The significance level adopted was 5%.

## RESULTS

This study included 83 ICNPs from a university hospital in Campo Grande, MS, Brazil, with a mean age of 41.3 ± 5.7 years, 78.3% of whom were women. The occurrence of lower back pain in the last 12 months was 72.3%, with variations between the Adult ICU (65.6%), Pediatric ICU (76.9%), and Neonatal ICU (76%).

Among participants, 27.7% reported no low back pain, while 45.8% reported recurrent episodes, defined as two or more episodes in the last 12 months, lasting at least one day, with a pain-free interval of 30 days or more between episodes. Acute pain was reported by 14.5%, subacute pain by 4.8%, and chronic pain by 7.2% of participants.

Table 1 presents the frequency distribution of lower back pain in the last 12 months according to sociodemographic, occupational, and clinical profile. Among nurses, the prevalence was 64.7%, while among nursing technicians it was 84.4%, with a trend towards a significant difference (p = 0.051).

**Table 1:** Distribution of low back pain occurrence in the last 12 months according to sociodemographic, occupational, and clinical profile, Campo Grande, Mato Grosso do Sul, Brazil, 2024 (N=83)

Variable		Lower back pain in the last 12 months				
		No		Yes		
		f	%	f	%	*p* value
Sex	Female	16	19.3	49	59.0	0.231
	Male	7	8.4	11	13.3	
Skin color	White	17	20.5	27	32.5	0.018
	Non-white	6	7.2	33	39.8	
Religion	No	1	1.2	7	8.4	0.434^*^
	Yes	22	26.5	53	63.9	
Monthly family income (R$)	≤ 22 thousand	22	26.5	57	68.7	1.000^*^
	> 22 thousand	1	1.2	3	3.6	
Position	Nurse	18	21.7	33	39.8	0.051
	Nursing technician	5	6	27	32.5	
Professional training level by category	Vocational training/undergraduate education	6	7.2	21	25.3	0.438
	Graduate education (lato and stricto sensu)	17	20.5	39	47	
Intensive Care Unit by category	Adult	11	13.3	21	25.3	0.283
	Infant (pediatric and neonatal)	12	14.5	39	47	
Weekly hours (h)	Up to 36	11	13.3	46	55.4	0.011
	> 36	12	14.5	14	16.9	
Intensive Care Unit experience (years)	Up to 10	7	8.4	29	34.9	0.141
	> 10	16	19.3	31	37.3	
Work schedule	Daytime	12	14.5	30	36.1	0.859
	Nighttime	11	13.3	30	36.1	
Other employment	No	12	14.5	47	56.6	0.019
	Yes	11	13.3	13	15.7	
Time spent sitting per weekday (h)	< 8	20	24.1	51	61.4	1.000^*^
	≥ 8	3	3.6	9	10.8	
Índice de Massa Corporal por categoria	< 25	7	8.9	19	24.1	0.764
	≥ 25	16	20.3	37	46.8	
Body Mass Index by category	Active	19	22.9	37	44.6	0.115^*^
	Slightly active	4	4.8	23	27.7	
Roland-Morris Disability Questionnaire; disability due to low back pain by category	Mild disability	23	27.7	56	67.5	0.572^*^
	Moderate/high disability	0	0.0	4	4.8	
Bianchi Stress Scale; occupational stress by category	Low	13	15.7	28	33.7	0.444
	Medium/high	10	12.0	32	38.6	

Note: p-values obtained by the chi-square test (χ^2^) or, where indicated (^*^), by Fisher’s exact test. Frequencies (f) and percentages (%) are presented for “low back pain yes” and “low back pain no”.

The prevalence of medium/high occupational stress was 50.6%, with variations between the Adult ICU (53.1%), Pediatric ICU (61.5%), and Neonatal ICU (36%). Among nurses, 63% reported medium/high stress levels, while 69% of nursing technicians reported low stress, a significant difference (p = 0.005).

Concerning the sociodemographic profile, 59% of women and 13.3% of men reported low back pain (p = 0.231). Skin color was significantly associated with low back pain, with a higher occurrence among non-whites than among whites, while there was no difference for religious belief or income.

In terms of occupational profile, the highest incidence of lower back pain was observed among professionals with a workload of up to 36 hours per week and no additional employment, but there was no significant difference for ICU categories or professional training.

Prolonged sitting and BMI did not show significant associations with the presence of low back pain. Analysis of the impact of low back pain on functional disability, according to the RMDQ, revealed that 44.6% of participants presented moderate to high disability, but there was no significant association between low back pain and functional disability or between low back pain and occupational stress.

In relation to physical activity (Table 2), there was no significant difference among individuals with and without low back pain for the variables analyzed.

**Table 2:** Association between physical activity levels and low back pain in intensive care nursing professionals: Mann-Whitney U test, Campo Grande, Mato Grosso do Sul, Brazil, 2024 (N=83)

Variable	Lower back pain in the last 12 months		*p* value
	No (n = 23)	Yes (n = 60)	
Walking (MET-min/week)	198 (495)	215 (668)	0.767
Moderate physical activity (MET-min/week)	240 (1280)	480 (915)	0.823
Vigorous physical activity (MET-min/week)	1140 (2640)	360 (1480)	0.077
Total physical activity (MET-min/week)	2640 (1355)	2190 (2705)	0.280
Total energy expenditure (Kcal-min/week)	3063 (2215)	2717 (3852)†	0.271

*Note: MET-min/week - Metabolic Equivalent of Task in minutes per week; Kcal-min/week - Total energy expenditure in kilocalories per minute over the week. Median and interquartile range values are presented as descriptive. †The “low back pain yes” group, for the Kcal-min/week variable, had a sample of 58 participants.*

As for physical fitness (Table 3), trunk flexor strength was significantly higher in the pain-free group than in the group with low back pain. Other physical fitness variables did not show significant differences among individuals with and without low back pain.

**Table 3:** Association between health-related physical fitness and low back pain in intensive care nursing professionals: t-test and Mann-Whitney U test, Campo Grande, Mato Grosso do Sul, Brazil, 2024 (N=79)

Variable	Lower back pain in the last 12 months		*p* value
	Não (n = 23)	Sim (n = 56)	
Body fat (%)	26.75 ±8.67	29.14 ±7.95	0.241
Sit-and-reach test (cm)	27.30 ±8.98	25.90 ±8.86	0.527
Handgrip strength (kgf)	36.00 (12.50)	35.00 (10.25)	0.901^*^
Ito test for trunk flexor endurance (s)	55.02 (28.33)	38.05 (24.07)	0.004^*^
Ito test for trunk extensor endurance (s)	92.50 (73.10)	82.61 (64.92)	0.359^*^
Ruffier-Dickson test (ind.)	8.52 ±2.12†	9.4 ±2.90†	0.212

Note:

* Mann-Whitney U test; †The TR-D (index) variable included 75 participants, of whom 54 belonged to the “low back pain yes” group, and 21 to the “low back pain no” group, after four participants did not perform the test due to transient health conditions, such as fatigue or discomfort, detected before the test was performed. Values are presented as mean and standard deviation (±) or median and interquartile range.

Table 4 presents physical fitness data comparing active (67.5%) and slightly active (32.5%) ICNPs. The analysis revealed that trunk flexor strength was greater in the active group compared to the slightly active group. There was no significant difference for the other variables assessed.

**Table 4:** Association between health-related physical fitness and physical activity levels in intensive care nursing professionals: t-test and Mann-Whitney U test, Campo Grande, Mato Grosso do Sul, Brazil, 2024 (N=79)

Variable	Ativo (n = 53)	Pouco ativo (n = 26)	*p* value
Body fat (%)	28.15 ±8.55	29.05 ±7.51	0.648
Sit-and-reach test (cm)	26.00 ±8.74	27.20 ±9.22	0.578
Handgrip strength (kgf)	34.00 (11.00)	36.00 (6.75)	0.316^*^
Ito test for trunk flexor strength (s)	49.00 (25.49)	38.12 (22.50)	0.049^*^
Ito test for trunk extensor strength (s)	78.70 (62.39)	93.3 (80.80)	0.456^*^
Ruffier-Dickson test (ind.)	8.83 ±2.56†	9.79 ±2.97†	0.150
Presence of discomfort or lower back pain in the last 12 months	37 (44.6%)	23 (27.7%)	0.115^**^

Note:

* Mann-Whitney U test;

** Fisher’s exact test; †The TR-D (index) variable included 75 participants, 50 from the “active” group and 25 from the “slightly active” group, after four participants did not perform the test due to transient health conditions, such as fatigue or discomfort, detected before the test. Values are presented as mean and standard deviation (±) or median and interquartile range.

## DISCUSSION

The prevalence of low back pain among ICNPs observed in this study was high, reaching 72.3% in total, with higher rates in the Pediatric ICU (76.9%) and Neonatal ICU (76%), and lower in the Adult ICU (65.6%). These data are similar to those of research conducted in a university hospital in the city of São Paulo, which revealed a prevalence of low back pain of 91.2% in the Adult ICU and 75% in the Pediatric ICU^(28)^. This elevated pattern of lower back pain in healthcare professionals, especially in ICUs, can be attributed to the intense physical demands of these settings, highlighting the occupational impact on musculoskeletal health.

Lower back pain was shown to be associated with sociodemographic factors, such as skin color, with a higher occurrence among non-white professionals compared to white professionals. This finding aligns with the literature, which associates racial disparities with a higher incidence of chronic lower back pain, especially in contexts of lower educational levels and adverse socioeconomic conditions, which increase the risk of persistent and disabling pain^(29)^. Educational level did not differ between individuals with and without low back pain (p > 0.05), suggesting that other sociodemographic and occupational factors may contribute to the prevalence of low back pain in ICNPs. In this study, low back pain was more prevalent among professionals with a workload of up to 36 hours per week and without additional employment. In this context, a study conducted with 85 ICU nurses showed that a high workload is associated with a significant increase in low back pain, with an Odds Ratio of 4.20^(30)^. In the present study, the higher occurrence of low back pain in ICNPs with a workload of up to 36 hours per week and no additional employment suggests that, for these professionals, the physical and emotional burden of their single job may be sufficient to develop low back pain. This highlights the importance of occupational factors, such as workload and employment status, in the onset of low back pain, even without an overload of overtime.

This scenario of overload, both physical and emotional, is also reflected in the prevalence of occupational stress, which was 50.6% in the present study, with higher levels among nurses (62.7%) compared to nursing technicians (31.3%). The association between job and stress was significant (p = 0.005), indicating greater exposure to stress among nurses. A study conducted at the University Hospital of Benha identified a prevalence of severe stress in 74% of the 100 nurses working in ICUs assessed. The high prevalence of stress reflects the emotional and physical overload common in intensive care settings, with negative impacts on nurses’ well-being and professional performance^(31)^.

In the present study, the assessment of physical fitness levels in ICNPs with and without lower back pain in the last 12 months identified greater trunk flexor muscle endurance in the pain-free group. There were no significant differences in other variables, including body composition, flexibility, muscle strength, trunk extensor muscle endurance, and cardiorespiratory endurance. The importance of trunk flexor muscle endurance, as observed in the present study, is supported by a previous study, which suggests that trunk muscle strength may play a crucial role in preventing low back pain. Individuals with weak trunk muscles were 2.5 times more likely to develop low back pain compared to individuals with normal muscle strength, highlighting the relevance of trunk flexors in protecting against this condition^(32)^. Another study confirmed that core strengthening exercises reduce lower back pain and increase the endurance of trunk flexors in nurses, reinforcing the benefits of muscle conditioning^(33)^. Active nurses had greater trunk flexor endurance compared to less active nurses (p = 0.049), with no differences in control group, flexibility, HGS, trunk extensor endurance, or cardiorespiratory capacity (p > 0.05), suggesting that regular physical activity is associated with greater trunk flexor endurance strength, possibly facilitating the performance of daily occupational tasks. These findings reinforce the importance of strengthening trunk flexor muscles to prevent low back pain. A randomized clinical trial with nurses demonstrated that an eight-week core stability training program significantly reduced nonspecific low back pain and disability, as well as improving trunk muscle strength, with benefits for performing occupational tasks in high-demand physical settings such as the ICU^(34)^. These exercises can help reduce work-related pain, improve posture, and alleviate occupational stress.

Finally, no significant differences were observed in physical activity levels in terms of walking, moderate or vigorous activities between professionals with and without low back pain. However, studies with larger samples have associated low back pain with a reduction in intense physical activity, indicating that pain can limit exercise, especially in groups with risk factors such as older age and low self-perceived health^(35)^. This discrepancy among the results indicates a complex relationship between pain and physical activity, in which additional factors may influence the continuity and intensity of exercise.

### Study limitations

A limitation of the study is its cross-sectional nature, which makes it impossible to determine causal relationships between physical fitness, low back pain, and occupational stress. Data collection at a single point in time also prevents the analysis of changes over time. Self-reported pain and occupational stress can introduce bias, since responses depend on memory, perception, or subjective aspects. Furthermore, recruitment at a single institution restricts the generalizability of results. Longitudinal studies are needed to assess occupational stress progression and its interactions with other variables. The BSS, although validated in Brazil, has limited evidence of international validation, restricting its comparability with global studies.

### Contributions to occupational health

The analysis highlights the importance of interventions that encourage an active lifestyle and muscle strengthening among ICNPs, given the relationship between low back pain and reduced trunk flexor strength, as well as the observed presence of occupational stress, without a statistically significant association with low back pain. Regular physical activity, along with a greater focus on strengthening trunk flexor muscles, has a potential protective effect and should be studied in more detail in this target population. Implementing health promotion programs, with periodic physical fitness assessments and resistance training, can help reduce pain, improve these professionals’ quality of life and well-being, and promote healthier work settings in the healthcare sector, positively impacting patient care quality.

## CONCLUSIONS

Occupational stress, although highly prevalent, was not associated with the occurrence of low back pain in ICNPs. Among the physical fitness components, trunk flexor strength stood out as a factor observationally associated with a lower occurrence of low back pain. Although the overall level of physical activity was not significantly related to low back pain, a trend, not statistically significant, of lower occurrence was observed among individuals who practice vigorous activities, suggesting that exercise intensity may positively influence spinal health in this population.

## Data Availability

The research data are available in a repository: https://doi.org/10.48331/scielodata.9AO6LV.
