# Supplementary material for: Low back pain, occupational stress, and physical fitness in intensive care nursing professionals
Source: Rev Bras Enferm. 2026 Aug 17;79:e20240583. doi: 10.1590/0034-7167-2024-0583 (PMC13481040; doi:10.1590/0034-7167-2024-0583)
Supplement: Translation

# Translation: Dor lombar, estresse ocupacional e aptidão física em profissionais de enfermagem de terapia intensiva

**DOI:** 10.1590/0034-7167-2024-0583pt

**Authors:** Ulisses Masseli Dias, Silvio Assis de Oliveira-Junior, Jeeser Alves de Almeida, Paula Felippe Martinez

**Affiliations:** I Universidade Federal de Mato Grosso do Sul. Campo Grande, Mato Grosso do Sul, Brasil; II Empresa Brasileira de Serviços Hospitalares. Campo Grande, Mato Grosso do Sul, Brasil

**Keywords:** Dor Lombar, Aptidão Física, Estresse Ocupacional, Enfermeiras e Enfermeiros, Unidades de Terapia Intensiva.

## Abstract

**Objetivos:**

avaliar a associação entre dor lombar, estresse ocupacional e aptidão física em profissionais de enfermagem de terapia intensiva.

**Métodos:**

estudo transversal quantitativo com amostragem por conveniência, incluindo dados sociodemográficos, estresse ocupacional e aptidão física (capacidade cardiorrespiratória, composição corporal, flexibilidade, força e resistência muscular). Dor lombar foi avaliada por escalas de incapacidade, duração e frequência.

**Resultados:**

entre 83 profissionais de enfermagem de terapia intensiva, 72,3% relataram dor lombar no último ano, e 50,6% apresentaram estresse ocupacional médio a alto. Dor lombar associou-se à carga horária e resistência dos flexores do tronco. As demais variáveis de aptidão física não variaram entre grupos. Incapacidade funcional foi semelhante entre indivíduos com e sem dor lombar.

**Conclusões:**

resistência dos flexores do tronco esteve associada à menor ocorrência de dor lombar em profissionais de enfermagem de terapia intensiva. Atividade física vigorosa apresentou tendência de associação, enquanto estresse ocupacional não apresentou relação com a dor lombar.

## INTRODUÇÃO

A dor lombar é uma condição prevalente entre profissionais de saúde, especialmente entre enfermeiros de Unidades de Terapia Intensiva (UTIs), com estudos revelando ocorrência entre 76% e 80,5%, frequentemente associados à alta carga laboral e às atividades fisicamente extenuantes^(1,2)^. Concomitantemente, o estresse ocupacional é frequente entre enfermeiros de UTI, predominando o nível moderado (87%), sendo menos comum em nível baixo (11,7%) ou alto (1,3%)^(3)^.

Em contrapartida, a aptidão física relacionada à saúde, definida como a capacidade de realizar atividades físicas diárias com vigor e sem fadiga excessiva, incluindo componentes como força muscular, capacidade cardiorrespiratória, flexibilidade e composição corporal^(4)^, emerge como um fator potencialmente protetor, influenciando positivamente a qualidade de vida e o bem-estar dos profissionais de saúde. A prática regular de atividade física tem sido associada à redução do estresse percebido e à melhoria do bem-estar mental em profissionais de saúde, embora mais estudos sejam necessários para elucidar a extensão desses benefícios^(5)^.

Componentes específicos da aptidão física, como força, resistência e flexibilidade, podem contribuir para a redução da dor lombar e do estresse ocupacional entre enfermeiros. O fortalecimento muscular e a flexibilidade auxiliam na prevenção de dores musculoesqueléticas, enquanto a resistência favorece a adaptação ao estresse físico e psicológico, promovendo maior resiliência no ambiente hospitalar^(6)^. Além disso, programas de exercícios que focam no fortalecimento muscular e na capacidade física podem reduzir a intensidade da dor lombar em profissionais de enfermagem, contribuindo para a prevenção de incapacidades ocupacionais^(7)^.

Apesar das evidências sobre os benefícios da aptidão física na mitigação da dor lombar e do estresse ocupacional, as relações específicas entre esses fatores em enfermeiros de UTI ainda são pouco exploradas, demandando estudos mais detalhados.

Compreender essas interações é essencial para o desenvolvimento de estratégias voltadas à promoção da saúde e do bem-estar dos profissionais de enfermagem de terapia intensiva (PETIs), fortalecendo tanto a segurança no ambiente de trabalho quanto a qualidade do cuidado prestado ao paciente. A identificação de fatores de risco e de proteção permite direcionar intervenções de forma mais precisa, contribuindo para a prevenção de distúrbios musculoesqueléticos e melhoria da qualidade de vida desses profissionais.

## OBJETIVOS

Avaliar a associação entre dor lombar, estresse ocupacional e aptidão física relacionada à saúde em PETIs ativos e pouco ativos de hospital universitário.

## MÉTODOS

### Aspectos éticos

O estudo foi conduzido em conformidade com os princípios éticos da Resolução nº 466/2012 do Conselho Nacional de Saúde e da Resolução CNS nº 580/2018, que regulamentam a anuência institucional e a não interferência na rotina dos serviços. A autorização institucional foi obtida por meio de resolução administrativa, que determina a citação do Hospital Universitário Maria Aparecida Pedrossian da Universidade Federal de Mato Grosso do Sul (HUMAP-UFMS) em todas as produções científicas do projeto. O projeto foi aprovado pelo Comitê de Ética em Pesquisa da UFMS, e todos os participantes assinaram o Termo de Consentimento Livre e Esclarecido.

### Desenho, período e local do estudo

Este estudo, de caráter observacional, analítico, transversal e quantitativo, utilizou amostragem não probabilística por conveniência, e foi reportado conforme as diretrizes *STrengthening the Reporting of OBservational studies in Epidemiology* ^(8)^. A coleta de dados foi realizada entre junho e julho de 2024 no HUMAP, localizado na região Centro-Oeste do Brasil, vinculado à UFMS e administrado pela Empresa Brasileira de Serviços Hospitalares (Ebserh).

### População e amostra; critérios de inclusão e exclusão

A população de estudo foi composta por 98 PETIs, formada por enfermeiros e técnicos de enfermagem atuantes nas UTI Adulto, UTI Pediátrica e UTI Neonatal de hospital universitário. Como toda a população-alvo foi analisada, o cálculo do tamanho da amostra não foi necessário. Os critérios de inclusão abrangeram PETIs em boas condições de saúde, com capacidade física para as atividades do estudo, com idade entre 18 e 55 anos e sem restrições quanto a sexo, cor ou raça/etnia. A triagem inicial foi realizada por meio de questionário clínico-ocupacional, destinado a avaliar o estado de saúde e a condição física, com itens sobre doenças, cirurgias e elegibilidade, e complementada por instrumentos validados: um questionário de atividade física; um questionário de incapacidade por dor lombar; e uma escala de estresse ocupacional. As boas condições de saúde e a capacidade física foram confirmadas por autorrelato no questionário inicial, que verificou a ausência de doenças sistêmicas, cirurgias na coluna, deficiências ou licenças médicas, bem como a aptidão para realizar os testes físicos sem contraindicações médicas, não sendo exigidos índices de normalidade. Foram excluídos PETIs com dor lombar associada a doenças sistêmicas, gestantes ou mulheres até seis meses pós-parto, histórico de cirurgia na coluna lombar, doenças articulares degenerativas, deficiências incapacitantes e aqueles em licença médica ou afastamento. Seis participantes foram excluídos com base nos critérios de exclusão definidos, e nove não concordaram em participar. A amostra foi composta por 51 enfermeiros e 32 técnicos de enfermagem, distribuídos entre as UTI Adulto, UTI Pediátrica e UTI Neonatal, com idades variando de 27 a 55 anos. No total, 79 PETIs completaram o estudo, enquanto quatro participantes não realizaram a segunda etapa, resultando em uma taxa de participação de 90,2%, conforme Figura 1.

**Figura 1:** Fluxograma de seleção de participantes para estudo com profissionais de enfermagem de terapia intensiva, Campo Grande, Mato Grosso do Sul, Brasil, 2024

*INSS - Instituto Nacional do Seguro Social.*

*Nota: todas as etapas do estudo foram concluídas por 79 participantes. Nove optaram por não participar; seis foram excluídos por inelegibilidade; e quatro não realizaram os testes físicos. Métricas: taxa de participação (90,2%) = [(83 / 92) × 100 ≈ 90,2%], baseada em participantes potenciais (n = 83) e elegíveis após triagem (n = 92); proporção de inclusão total (85,9%) = [(79 / 92) × 100 ≈ 85,9%] baseada nos que completaram o estudo (n = 79) e elegíveis após triagem (n = 92).*

### Protocolo e variáveis do estudo

A coleta de dados foi realizada em duas etapas: (1) Questionário Sociodemográfico, Ocupacional e Clínico (QSOC), aplicado *online* (*Google Forms* ^®^) abordando dados sociodemográficos, ocupacionais e clínicos, incluindo o Questionário Internacional de Atividade Física (IPAQ)^(9)^, o Questionário de Incapacidade de Roland-Morris (QRM)^(10)^ e a Escala Bianchi de Estresse (EBS)^(11)^; e (2) realização de avaliações da aptidão física, em sessões agendadas, destinadas à coleta de dados de saúde e aptidão física, contemplando o Índice de Massa Corporal (IMC)^(12)^, o percentual de gordura corporal (GC) pelo método das sete dobras de Jackson e Pollock^(13)^, a força de preensão manual (FPM)^(12)^, a flexibilidade pelo teste de sentar e alcançar (TSA)^(14)^, a força de resistência dos músculos do tronco pelo teste de Ito^(15)^ e a avaliação cardiorrespiratória pelo teste de Ruffier-Dickson (TRD)^(16)^.

O QSOC coletou informações sobre sexo, idade, cor/raça, crença religiosa, nível educacional, renda familiar, cargo, período (turno) e jornada semanal de trabalho, tempo de experiência em enfermagem geral e em enfermagem intensivista, existência de outro vínculo empregatício, e atividade associada. Também foram avaliadas a duração e frequência da dor lombar nos últimos 12 meses, sendo sua presença classificada em cinco grupos, nomeadamente ausência de dor, episódios recorrentes, dor aguda (≤ 6 semanas), dor subaguda (6-12 semanas) e dor crônica (> 12 semanas), com base na duração e na frequência^(17)^. Para análise estatística, a dor lombar foi dicotomizada em presença (qualquer relato nos últimos 12 meses) e ausência (sem dor).

A incapacidade relacionada à dor lombar foi medida pelo QRM^(10,18)^, cuja pontuação varia de 0 (sem incapacidade) a 24 (incapacidade grave). Para a estratificação da gravidade, adotou-se a categorização em leve (0-8), moderada (9-16) e alta (17-24)^(19)^. Para fins analíticos, a incapacidade funcional foi dicotomizada em leve (≤ 8 pontos) e moderada/alta (> 8 pontos), buscando maior clareza interpretativa e consistência estatística.

O estresse ocupacional foi analisado pela EBS^(11)^, com 51 itens, organizados em seis domínios, utilizando o escore total, dicotomizado ponto de corte ≤ 3, para “baixo”, e > 3, para “médio/alto”.

Adicionalmente, o nível de atividade física foi avaliado pelo IPAQ, versão curta, validada no Brasil^(9)^. O IPAQ analisa o tempo dedicado a caminhadas, atividades moderadas, vigorosas e comportamento sedentário. Com base nos resultados, os indivíduos foram categorizados em dois grupos: ativo, que inclui as categorias “muito ativo” e “ativo”, com níveis adequados de atividade física; e pouco ativo, que engloba as categorias “insuficientemente ativo” e “sedentário”, que representam níveis insuficientes de atividade física ou ausência de envolvimento regular em exercícios. O IMC foi calculado pela razão entre massa corporal (kg) e estatura ao quadrado (m^2^)^(12)^. No estudo, a massa corporal foi medida com balança eletrônica G-Tech (Glass 3FWB, precisão de 100 g), e a estatura, com estadiômetro digital portátil da marca BIC (precisão de 0,1 cm)^(20)^. A classificação do IMC foi dicotomizada em IMC ≥ 25 kg/m^2^ (risco elevado) e IMC < 25 kg/m^2^ (risco baixo).

Quanto aos componentes da aptidão física relacionados à saúde, a composição corporal, estimada pelo percentual de GC, que constitui um indicador de risco para doenças metabólicas, cardiovasculares e psicossociais^(21)^, foi avaliada pela espessura de dobras cutâneas em sete pontos anatômicos com o adipômetro científico *Opus Max*, sendo calculada pelas fórmulas de Jackson e Pollock (1978)^(13)^. A FPM, indicador da força muscular global^(22)^ e variável associada à presença de dor lombar^(23)^, foi avaliada com o participante sentado, considerando a força de preensão da mão dominante, medida com dinamômetro hidráulico Jamar, em três tentativas, com intervalo de um minuto entre elas^(12)^, sendo os resultados comparados com classificações por sexo e faixa etária^(24)^. O TSA, que avalia a flexibilidade da cadeia posterior^(13)^, foi realizado com o participante sentado sobre superfície estável revestida por colchonete fino (1,5 cm de espessura), com joelhos estendidos e pés fixos sem calçados, flexionando o tronco à frente até alcançar a maior distância possível com os dedos. Utilizando um banco de Wells portátil (*Prime Med*), o teste foi realizado em três tentativas, considerando-se a maior distância alcançada a partir do dedo médio, e os resultados foram comparados com a classificação de Pollock ML e Wilmore JH (1993)^(25)^. A força de resistência dos músculos flexores e extensores do tronco foi avaliada pelo teste de Ito^(15)^, que utiliza posturas isométricas de flexão e extensão realizadas em decúbito ventral e dorsal até o limite de resistência do participante (máximo de cinco minutos). O tempo foi registrado com cronômetro digital.

Por fim, a aptidão cardiorrespiratória foi avaliada pelo TRD, que consiste em realizar 30 agachamentos em 45 segundos^(16,20,26,27)^, sendo um teste validado clinicamente e associado à redução de riscos cardiovasculares e ao aumento do VO_2_ máx^(26,27)^. A postura foi ajustada para garantir a flexão de 90° dos joelhos, e a frequência cardíaca (P1, P2 e P3) foi aferida com o *smartwatch Samsung Gear S3 Frontier*. O ritmo dos 30 agachamentos foi controlado a 40 movimentos por minuto (mpm) pelo metrônomo do aplicativo *Soundcorset Tuner & Metronome* ^®^, e os resultados foram interpretados pelo Índice de Ruffier^(20,27)^.

### Análise estatística

Os dados coletados via *Google Forms* ^®^ foram analisados no *software* Jamovi, versão 2.6 (Sydney, Austrália). Foram apresentados em medidas descritivas, incluindo média e desvio padrão, mediana e amplitude interquartílica, além de frequências absolutas e relativas. Variáveis categóricas foram avaliadas pelo teste exato de Fisher ou teste do qui-quadrado (χ^2^), enquanto a normalidade das variáveis contínuas foi analisada pelo teste de Shapiro-Wilk. A associação entre aptidão física e nível de atividade física foi testada comparando os grupos ativos e pouco ativos, utilizando teste t de Student para variáveis com distribuição normal e teste de Mann-Whitney para variáveis com distribuição não paramétrica. O nível de significância adotado foi de 5%.

## RESULTADOS

Este estudo incluiu 83 PETIs de hospital universitário em Campo Grande, MS, Brasil, com média de idade de 41,3 ± 5,7 anos, sendo 78,3% mulheres. A ocorrência de dor lombar nos últimos 12 meses foi de 72,3%, com variações entre as UTI Adulto (65,6%), UTI Pediátrica (76,9%) e UTI Neonatal (76%).

Entre os participantes, 27,7% relataram ausência de dor lombar, enquanto 45,8% apresentaram episódios recorrentes, definidos como dois ou mais episódios nos últimos 12 meses, com duração de pelo menos um dia e intervalo sem dor de 30 dias ou mais entre os episódios. A dor aguda foi reportada por 14,5%, a subaguda, por 4,8%, e a crônica, por 7,2% dos participantes.

Na Tabela 1, apresenta-se a distribuição de frequência de dor lombar nos últimos 12 meses segundo o perfil sociodemográfico, ocupacional e clínico. Entre os enfermeiros, a prevalência foi de 64,7%, enquanto entre técnicos de enfermagem foi de 84,4%, com tendência à diferença significativa (p = 0,051).

**Tabela 1:** Distribuição da ocorrência de dor lombar nos últimos 12 meses segundo perfil sociodemográfico, ocupacional e clínico, Campo Grande, Mato Grosso do Sul, Brasil, 2024 (N=83)

Variável		Dor lombar nos últimos 12 meses				
		Não		Sim		
		f	%	f	%	Valor de *p*
Sexo	Feminino	16	19,3	49	59,0	0,231
	Masculino	7	8,4	11	13,3	
Cor da pele	Branca	17	20,5	27	32,5	0,018
	Não branca	6	7,2	33	39,8	
Crença religiosa	Não	1	1,2	7	8,4	0,434^*^
	Sim	22	26,5	53	63,9	
Renda familiar mensal (R$)	≤ 22 mil	22	26,5	57	68,7	1,000^*^
	> 22 mil	1	1,2	3	3,6	
Cargo	Enfermeiro	18	21,7	33	39,8	0,051
	Técnico de enfermagem	5	6	27	32,5	
Nível de formação profissional por categoria	Ensino técnico/graduação	6	7,2	21	25,3	0,438
	Pós-graduação (*lato* e *stricto sensu*)	17	20,5	39	47	
Unidade de Terapia Intensiva por categoria	Adulto	11	13,3	21	25,3	0,283
	Infantil (pediátrica e neonatal)	12	14,5	39	47	
Jornada semanal (h)	Até 36	11	13,3	46	55,4	0,011
	> 36	12	14,5	14	16,9	
Experiência em Unidade de Terapia Intensiva (anos)	Até 10	7	8,4	29	34,9	0,141
	> 10	16	19,3	31	37,3	
Período de trabalho	Diurno	12	14,5	30	36,1	0,859
	Noturno	11	13,3	30	36,1	
Outro vínculo empregatício	Não	12	14,5	47	56,6	0,019
	Sim	11	13,3	13	15,7	
Tempo sentado por dia de semana (h)	< 8	20	24,1	51	61,4	1,000^*^
	≥ 8	3	3,6	9	10,8	
Índice de Massa Corporal por categoria	< 25	7	8,9	19	24,1	0,764
	≥ 25	16	20,3	37	46,8	
Nível de atividade física por categoria	Ativo	19	22,9	37	44,6	0,115^*^
	Pouco ativo	4	4,8	23	27,7	
Questionário de Incapacidade de Roland-Morris; incapacidade por dor lombar por categoria	Incapacidade leve	23	27,7	56	67,5	0,572^*^
	Incapacidade moderada/alta	0	0,0	4	4,8	
Escala Bianchi de Estresse; estresse ocupacional por categoria	Baixo	13	15,7	28	33,7	0,444
	Médio/alto	10	12,0	32	38,6	

Nota: valores de p obtidos pelo teste qui-quadrado (χ^2^) ou, onde indicado (^*^), pelo teste exato de Fisher. Frequências (f) e percentuais (%) apresentadas para “dor lombar sim” e “dor lombar não”.

A prevalência de estresse ocupacional médio/alto foi de 50,6%, com variações entre as UTI Adulto (53,1%), UTI Pediátrica (61,5%) e UTI Neonatal (36%). Entre enfermeiros, 63% relataram níveis médios/altos de estresse, enquanto 69% dos técnicos de enfermagem relataram estresse baixo, com diferença significativa (p = 0,005).

Quanto ao perfil sociodemográfico, 59% das mulheres e 13,3% dos homens relataram dor lombar (p = 0,231). A cor da pele esteve significativamente associada à dor lombar, sendo maior a ocorrência entre não brancos do que entre brancos, enquanto não houve diferença para crença religiosa ou renda.

No perfil ocupacional, a maior ocorrência de dor lombar foi observada entre profissionais com carga horária de até 36 horas semanais e sem vínculo empregatício adicional, mas não houve diferença significativa para as categorias de UTI ou formação profissional.

A permanência na posição sentada e o IMC não apresentaram associações significativas com a presença de dor lombar. A análise do impacto da dor lombar sobre a incapacidade funcional, conforme o QRM, revelou que 44,6% dos participantes apresentaram incapacidade moderada a alta, mas não houve associação significativa entre a dor lombar e a incapacidade funcional ou entre a dor lombar e o estresse ocupacional.

Em relação à atividade física (Tabela 2), não houve diferença significativa entre os indivíduos com e sem dor lombar para as variáveis analisadas.

**Tabela 2:** Associação entre níveis de atividade física e dor lombar em profissionais de enfermagem de terapia intensiva: teste U de Mann-Whitney, Campo Grande, Mato Grosso do Sul, Brasil, 2024 (N=83)

Variável	Dor lombar nos últimos 12 meses		Valor de *p*
	Não (n = 23)	Sim (n = 60)	
Caminhada (MET-min/semana)	198 (495)	215 (668)	0,767
Atividade física moderada (MET-min/semana)	240 (1280)	480 (915)	0,823
Atividade física vigorosa (MET-min/semana)	1140 (2640)	360 (1480)	0,077
Atividade física total (MET-min/semana)	2640 (1355)	2190 (2705)	0,280
Gasto energético total (Kcal-min/semana)	3063 (2215)	2717 (3852)†	0,271

*Nota: MET-min/semana - Equivalente Metabólico de Tarefa em minutos por semana; Kcal-min/semana - Gasto energético total em quilocalorias por minuto ao longo da semana. Valores de mediana e amplitude interquartílica são apresentados como descritivos. †O grupo “dor lombar sim”, para a variável Kcal-min/semana, contou com uma amostra de 58 participantes.*

Concernente à aptidão física (Tabela 3), a resistência dos flexores do tronco foi significativamente maior no grupo sem dor do que no grupo com dor lombar. Outras variáveis de aptidão física não apresentaram diferenças significativas entre os indivíduos com e sem dor lombar.

**Tabela 3:** Associação entre aptidão física relacionada à saúde e dor lombar em profissionais de enfermagem de terapia intensiva: teste t e teste U de Mann-Whitney, Campo Grande, Mato Grosso do Sul, Brasil, 2024 (N=79)

Variável	Dor lombar nos últimos 12 meses		Valor de *p*
	Não (n = 23)	Sim (n = 56)	
Gordura corporal (%)	26,75 ±8,67	29,14 ±7,95	0,241
Teste de sentar e alcançar (cm)	27,30 ±8,98	25,90 ±8,86	0,527
Força de preensão manual (kgf)	36,00 (12,50)	35,00 (10,25)	0,901^*^
Teste de Ito para resistência dos flexores do tronco (s)	55,02 (28,33)	38,05 (24,07)	0,004^*^
Teste de Ito para resistência dos extensores do tronco (s)	92,50 (73,10)	82,61 (64,92)	0,359^*^
Teste de Ruffier-Dickson (índ.)	8,52 ±2,12†	9,4 ±2,90†	0,212

Nota:

* Teste U de Mann-Whitney; †A variável TR-D (índ.) incluiu 75 participantes, dos quais 54 pertenciam ao grupo “dor lombar sim”, e 21, ao grupo “dor lombar não”, após quatro participantes não realizaram o teste devido a condições de saúde transitórias, como cansaço ou desconforto, detectadas antes da realização do teste. Os valores estão apresentados como média e desvio padrão (±) ou mediana e amplitude interquartílica.

Na Tabela 4, são apresentados os dados de aptidão física comparando PETIs ativos (67,5%) e pouco ativos (32,5%). A análise revelou que a resistência dos flexores do tronco foi maior no grupo de indivíduos ativos em comparação aos pouco ativos. Não houve diferença significativa para as demais variáveis avaliadas.

**Tabela 4:** Associação entre aptidão física relacionada à saúde e níveis de atividade física em profissionais de enfermagem de terapia intensiva: teste t e teste U de Mann-Whitney, Campo Grande, Mato Grosso do Sul, Brasil, 2024 (N=79)

Variável	Ativo (n = 53)	Pouco ativo (n = 26)	Valor de *p*
Gordura corporal (%)	28,15 ±8,55	29,05 ±7,51	0,648
Teste de sentar e alcançar (cm)	26,00 ±8,74	27,20 ±9,22	0,578
Força de preensão manual (kgf)	34,00 (11,00)	36,00 (6,75)	0,316^*^
Teste de Ito para resistência dos flexores do tronco (s)	49,00 (25,49)	38,12 (22,50)	0,049^*^
Teste de Ito para resistência dos extensores do tronco (s)	78,70 (62,39)	93,3 (80,80)	0,456^*^
Teste de Ruffier-Dickson (índ.)	8,83 ±2,56†	9,79 ±2,97†	0,150
Presença de desconforto ou dor lombar nos últimos 12 meses	37 (44,6%)	23 (27,7%)	0,115^**^

Nota:

* Teste U de Mann-Whitney;

** Teste exato de Fisher; †A variável TR-D (índ.) contou com 75 participantes, sendo 50 do grupo “ativo”, e 25, do grupo “pouco ativo”, após quatro participantes não realizaram o teste devido a condições de saúde transitórias, como cansaço ou desconforto, detectadas antes da realização do teste. Os valores estão apresentados como média e desvio padrão (±) ou mediana e amplitude interquartílica.

## DISCUSSÃO

A prevalência de dor lombar entre os PETIs observada neste estudo foi alta, atingindo 72,3% no total, com maiores taxas nas UTI Pediátrica (76,9%) e UTI Neonatal (76%), e menor na UTI Adulto (65,6%). Esses dados são semelhantes aos de pesquisa realizada em hospital universitário na cidade de São Paulo, que revelou prevalência de dor lombar de 91,2% na UTI Adulto e 75% na UTI Pediátrica^(28)^. Esse padrão elevado de dor lombar em profissionais de saúde, sobretudo em UTI, pode ser atribuído às exigências físicas intensas desses ambientes, evidenciando o impacto ocupacional na saúde musculoesquelética.

A dor lombar se mostrou associada a fatores sociodemográficos, como cor da pele, com maior ocorrência entre profissionais não brancos em relação aos brancos. Tal achado se alinha à literatura, que associa disparidades raciais à maior incidência de dor lombar crônica, especialmente em contextos de menor nível educacional e condições socioeconômicas adversas, que aumentam o risco de dor persistente e incapacitante^(29)^. O nível educacional não diferiu entre os indivíduos com e sem dor lombar (p > 0,05), sugerindo que outros fatores sociodemográficos e ocupacionais podem contribuir para a prevalência de dor lombar em PETIs. Neste estudo, a dor lombar foi mais prevalente entre profissionais com carga horária de até 36 horas semanais e sem vínculo empregatício adicional. Neste contexto, estudo realizado com 85 enfermeiros de UTI mostrou que uma carga de trabalho elevada está associada ao aumento significativo da dor lombar, com *Odds Ratio* de 4,20^(30)^. No presente estudo, a maior ocorrência de dor lombar em PETIs com carga horária de até 36 horas semanais e sem vínculo empregatício adicional sugere que, para esses profissionais, a carga física e emocional do único emprego pode ser suficiente para o desenvolvimento da dor lombar. Isso destaca a importância de fatores ocupacionais, como a carga de trabalho e o vínculo empregatício, no surgimento de dor lombar, mesmo sem uma sobrecarga de horas extras.

Esse cenário de sobrecarga, tanto física quanto emocional, também é refletido na prevalência de estresse ocupacional, que foi de 50,6% no presente estudo, com níveis mais elevados entre enfermeiros (62,7%) em relação aos técnicos de enfermagem (31,3%). A associação entre cargo e estresse foi significativa (p = 0,005), indicando maior exposição ao estresse entre enfermeiros. Estudo realizado no Hospital Universitário de Benha identificou prevalência de estresse severo em 74% dos 100 enfermeiros atuantes em UTIs avaliados. A alta prevalência de estresse reflete a sobrecarga emocional e física comum em ambientes de terapia intensiva, com impactos negativos no bem-estar e na performance profissional dos enfermeiros^(31)^.

No presente estudo, a avaliação dos níveis de aptidão física em PETIs com e sem dor lombar nos últimos 12 meses identificou maior resistência muscular dos flexores do tronco no grupo sem dor. Não houve diferenças significativas em outras variáveis, incluindo composição corporal, flexibilidade, força muscular, resistência dos músculos extensores do tronco e resistência cardiorrespiratória. A importância da resistência muscular dos flexores do tronco, como observada no presente estudo, é corroborada por estudo anterior, que sugere que a força muscular do tronco pode desempenhar um papel crucial na prevenção da dor lombar. Indivíduos com fraqueza nos músculos do tronco apresentaram 2,5 vezes mais chances de desenvolver dor lombar em comparação aos indivíduos com força muscular normal, destacando a relevância dos flexores do tronco na proteção contra essa condição^(32)^. Outro estudo confirmou que exercícios de fortalecimento do core reduzem a dor lombar e aumentam a resistência dos flexores do tronco em enfermeiros, reforçando os benefícios do condicionamento muscular^(33)^. Enfermeiros ativos tiveram maior resistência dos flexores do tronco em comparação aos pouco ativos (p = 0,049), sem diferenças em GC, flexibilidade, FPM, resistência dos extensores do tronco ou capacidade cardiorrespiratória (p > 0,05), sugerindo que a atividade física regular está associada à maior força de resistência dos flexores do tronco, possivelmente facilitando a execução de tarefas ocupacionais diárias. Esses achados reforçam a importância de fortalecer os músculos flexores do tronco para prevenir a dor lombar. Ensaio clínico randomizado com enfermeiros demonstrou que um programa de treinamento de estabilidade do core de oito semanas reduziu significativamente a dor lombar inespecífica e a incapacidade, além de melhorar a força muscular do tronco, com benefícios para a execução de tarefas ocupacionais em ambientes de alta demanda física, como UTI^(34)^. Esses exercícios podem contribuir para a redução de dores relacionadas ao trabalho, melhoria da postura e alívio do estresse ocupacional.

Por fim, não foram observadas diferenças significativas nos níveis de atividade física em termos de caminhada, atividades moderadas ou vigorosas entre profissionais com e sem dor lombar. Entretanto, estudos com amostras maiores associaram a dor lombar à redução de atividades físicas intensas, indicando que a dor pode limitar o exercício, especialmente em grupos com fatores de risco, como idade mais avançada e baixa percepção de saúde^(35)^. Essa discrepância entre os resultados indica uma relação complexa entre dor e prática de atividade física, em que fatores adicionais podem influenciar a continuidade e intensidade do exercício.

### Limitações do estudo

Uma limitação do estudo é sua natureza transversal, que impossibilita a determinação de relações causais entre aptidão física, dor lombar e estresse ocupacional. A coleta de dados em um único momento também impede a análise de alterações ao longo do tempo. O autorrelato de dor e estresse ocupacional podem introduzir viés, uma vez que as respostas dependem da memória, da percepção ou de aspectos subjetivos. Ademais, o recrutamento em uma única instituição restringe a generalização dos resultados. Estudos longitudinais são necessários para avaliar a progressão do estresse ocupacional e suas interações com outras variáveis. A EBS, embora validada no Brasil, possui evidências limitadas de validação internacional, restringindo sua comparabilidade com estudos globais.

### Contribuições para a área da saúde ocupacional

A análise realizada destaca a importância de intervenções que incentivem um estilo de vida ativo e o fortalecimento muscular entre os PETIs, diante da relação entre dor lombar e menor resistência dos flexores do tronco, bem como da presença observada de estresse ocupacional, sem associação estatística com a dor lombar. A prática regular de atividades físicas, juntamente com um maior enfoque no fortalecimento da musculatura flexora do tronco, tem um potencial efeito protetor, devendo ser mais detalhadamente estudada nesta população-alvo. Implementar programas de promoção da saúde, com avaliações periódicas de aptidão física e treinamento de resistência, pode ajudar a reduzir a dor, melhorar a qualidade de vida e o bem-estar desses profissionais, além de promover ambientes de trabalho mais saudáveis no setor de saúde, refletindo positivamente na qualidade do atendimento ao paciente.

## CONCLUSÕES

O estresse ocupacional, embora de alta prevalência, não apresentou associação com a ocorrência de dor lombar em PETIs. Entre os componentes da aptidão física, a resistência dos flexores do tronco se destacou como fator associado observacionalmente à menor ocorrência de dor lombar. Embora o nível geral de atividade física não tenha se mostrado significativamente relacionado à dor lombar, observou-se uma tendência, não estatisticamente significativa, de menor ocorrência entre indivíduos que praticam atividades vigorosas, sugerindo que a intensidade do exercício pode influenciar positivamente a saúde da coluna nessa população.

## Data Availability

Os dados de pesquisa estão disponíveis em repositório: https://doi.org/10.48331/scielodata.9AO6LV.
